# Supplementary material for: Predicting the Burden for Surgical Aortic Valve Replacement in a Tertiary Centre: The Impact of Aged Populations for the Next Decades
Source: J Clin Med. 2025 May 12;14(10):3365. doi: 10.3390/jcm14103365 (PMC12111943; doi:10.3390/jcm14103365)
Supplement: Supplementary file 1 [file jcm-14-03365-s001.zip › jcm-3613506-supplementary/Supplementary Table s1.pdf]

Supplementary Table s1 – Referral area of the Cardiothoracic Surgery Department

Region	Hospital	Municipalities
Lisboa and Vale do Tejo	Hospital de Santa Maria, EPE	• Lisboa
	Hospital Distrital de Santarém, EPE (14)	• Almeirim<br>• Alpiarça<br>• Cartaxo<br>• Chamusca<br>• Coruche<br>• Golegã<br>• Salvaterra de Magos<br>• Santarém<br>• Rio Maior
	Centro Hospitalar Barreiro/Montijo, EPE (15)	• Barreiro<br>• Moita<br>• Montijo<br>• Alcochete
	Hospital de Loures, PPP (16)	• Odivelas<br>• Sobral de Monte Agraço<br>• Mafra[1]<br>• Loures[2]
	Hospital Garcia de Orta, EPE (16)	• Almada<br>• Seixal
Alentejo	Unidade Local de Saúde do Litoral Alentejano, EPE (17)	• Alcácer do Sal<br>• Grândola<br>• Odemira<br>• Santiago do Cacém<br>• Sines
Algarve	Centro Hospitalar do Algarve, EPE (18)	• Albufeira<br>• Alcoutim<br>• Aljezur<br>• Castro Marim<br>• Faro<br>• Lagoa<br>• Lagos<br>• Loulé<br>• Monchique<br>• Olhão<br>• Portimão<br>• São Brás de Alportel<br>• Silves<br>• Tavira<br>• Vila do Bispo<br>• Vila Real de Santo António

1 Mafra only refers patients to the Hospital de Loures, PPP, from 4 out of 17 parishes in the municipality (Malveira, Milharado, Santo Estevão das Galés, and Venda do Pinheiro).

2 Loures only refers patients to the Hospital de Loures, PPP, from 11 out of 18 parishes in the municipality (Apelação, Bucelas, Camarate, Fanhões, Frielas, Loures, Lousa, Unhos, Santo António dos Cavaleiros, Santo Antão do Tojal, and São Julião do Tojal).
