# Supplementary material for: Predicting the Burden for Surgical Aortic Valve Replacement in a Tertiary Centre: The Impact of Aged Populations for the Next Decades
Source: J Clin Med. 2025 May 12;14(10):3365. doi: 10.3390/jcm14103365 (PMC12111943; doi:10.3390/jcm14103365)
Supplement: Supplementary file 1 [file jcm-14-03365-s001.zip › jcm-3613506-supplementary/Supplementary Table s2.pdf]

Supplementary Table s2 – Demographic evolution of the population under study from 2001 to 2021

	2001			2011			2021		
	Total	Male	Female	Total	Male	Female	Total	Male	Female
**TOTAL POPULATION**		**1 042 214**	**1 125 005**		**1 084 132**	**1 191 235**		**1 103 886**	**1 215 430**
**0-4 YEARS**		53 952	51 161		60 699	57 870		54 103	51 922
**5-9 YEARS**		51 518	48 499		58 620	54 640		53 968	51 121
**10-14 YEARS**		53 578	51 044		55 882	53 675		58 881	56 673
**15-19 YEARS**		64 722	62 352		55 041	53 361		59 648	56 661
**20-24 YEARS**		81 792	79 732		59 267	58 357		61 786	58 981
**25-29 YEARS**		85 947	82 916		70 271	72 142		64 749	63 593
**30-34 YEARS**		75 368	74 109		83 730	87 318		68 276	68 308
**35-39 YEARS**		73 005	75 389		88 172	90 655		74 479	77 084
**40-44 YEARS**	**2 167 219**	72 083	76 041	**2 275 367**	76 439	79 285	**2 319 316**	84 131	89 740
**45-49 YEARS**		70 977	75 950		73 309	78 341		85 848	92 323
**50-54 YEARS**		71 712	77 031		71 474	78 315		74 129	81 723
**55-59 YEARS**		64 711	71 319		68 912	77 336		69 635	80 315
**60-64 YEARS**		60 411	67 553		68 314	77 723		66 533	78 812
**65-69 YEARS**		55 881	68 022		59 246	70 397		62 982	75 200
**70-74 YEARS**		45 116	59 780		50 346	62 441		59 488	72 145
**75-79 YEARS**		32 900	48 105		40 413	57 341		45 816	59 990
**80-84 YEARS**		17 325	29 591		26 301	43 600		32 843	47 378
**>85 YEARS**		11 216	26 411		17 696	38 438		26 591	53 461
